# Toxic Elements in Honeys of Different Geographical Origin: From Poland Versus from Algeria

**DOI:** 10.3390/molecules31101620

**Published:** 2026-05-12

**Authors:** Nessrine Kazi-Tani, Anna Puścion-Jakubik, Hocine Allali, Nadia Aissaoui, Katarzyna Socha

**Affiliations:** 1 Laboratory of Organic Electrolytes and Polyelectrolytes Application (LAEPO), Abou Bekr Belkaïd University, P.O. Box 119, Tlemcen 13000, Algeria; nessrine.kazitani@univ-tlemcen.dz; 2 Department of Bromatology, Faculty of Pharmacy with the Division of Laboratory Medicine, Medical University of Białystok, Mickiewicza 2D Street, 15-222 Bialystok, Poland; katarzyna.socha@umb.edu.pl; 3 Spectrochemistry and Structural Pharmacology Laboratory (LSPS), Department of Chemistry, Faculty of Sciences, Abou Bekr Belkaïd University, P.O. Box 119, Tlemcen 13000, Algeria; 4 Laboratory of the Sustainable Management of Natural Resources in Arid and Semi-Arid Areas (GDRN), Faculty of Natural Sciences and Life, University of Naama, P.O. Box 66, Naama 45000, Algeria; aissaoui.nadia@cuniv-naama.dz

**Keywords:** mercury, cadmium, lead, arsenic, safety

## Abstract

Natural honey is a widely consumed product in various cultures. Owing to the foraging behaviour of bees and the unique production process of honey, this matrix serves as a critical tool for environmental biomonitoring. The aim of this study was to assess the content of selected toxic elements, specifically cadmium (Cd), lead (Pb), arsenic (As), and mercury (Hg), in honeys from Poland and Algeria. Additionally, the existence of health risks associated with long-term consumption was assessed. The As, Cd, and Pb content was determined using ICP-MS after prior microwave digestion of the samples in a closed system. Hg content was determined directly using the AAS method with the amalgamation technique. A total of 41 honey samples from Algeria and Poland were analysed. The content of the tested toxic elements in most samples was low. The results obtained for Pb were compared to applicable standards; exceedances for Pb were observed in 7% of the samples. Despite this, the estimated exposure did not indicate a significant health risk to consumers. The above data indicate that honey is not only a valuable nutritional product but also represents an important bioindicator of environmental pollution.

## 1. Introduction

Honey is a natural product produced by bees from nectar or honeydew, subjected to enzymatic processes [1]. It is an essential component of sustainable agriculture, vital for human well-being. Its chemical composition—particularly the content of toxic elements—can act as an indirect indicator of environmental quality [2]. The complex composition of honey includes sugars such as glucose, fructose, and sucrose, as well as numerous organic compounds, including organic acids, enzymes, amino acids (such as proline), vitamins, phenolic compounds, and volatile substances responsible for sensory properties [3,4,5]. The natural mineral content, typically ranging from 0.04% to 0.2%, is primarily determined by its botanical origin [6,7], resulting in a diversity of mineral elements that is often used to determine the geographical and botanical origin of different honey varieties [8]. The essential trace elements contained in honey are crucial for many physiological processes [9,10]. Although heavy metals occur naturally, their concentration in this natural product is increasing due to intensified anthropogenic activities, such as industrial emissions and the widespread use of agrochemicals. The presence of toxic elements in honey therefore poses a serious threat to public health after long-term exposure [11,12,13,14,15]. Given their persistence in the environment and tendency to accumulate in vital human organs, toxic elements enter the food chain and pose a significant health risk [16]. Therefore, regulatory frameworks, exemplified by the European Union’s establishment of a maximum permissible concentration (MPC) of lead (Pb) in honey of 100 µg/kg [17], emphasise the need for rigorous monitoring of the quality of this product. Metal content in honey is a holistic reflection of local environmental conditions and geological features [18,19]. The correlation between heavy metal concentrations and characteristics such as colour (e.g., darker honeys typically contain higher levels of macro- and micronutrients) further enhances the usefulness of honey analysis for assessing environmental quality [6,9].

Although numerous studies have assessed metal contamination in honey regionally, a direct quantitative assessment comparing and contrasting toxic metal burdens between North African sources and established European reference sources, integrating standardised toxicological risk indices, is not known. This study addresses this scientific gap by establishing a comparative framework that is essential, as honey’s trace element profile is not merely a reflection of botanical origin but serves as a high-fidelity proxy for regional geochemical signatures and anthropogenic atmospheric deposition [20,21,22]. By juxtaposing North African and Central European matrices, this study elucidates how divergent industrial legacies and agricultural practices manifest in the food chain, thereby providing critical data for global food safety harmonisation and environmental biomonitoring strategies [23,24]. The novelty of this research lies in its transcontinental approach, offering a broader perspective on the global relevance of honey as a bioindicator.

Given this identified scientific gap and the growing need to monitor food quality across geographic boundaries, this study aimed to conduct a comparative analysis. The primary goal was to determine the levels of toxic elements in Algerian honey samples (from northern Algeria) of diverse botanical origins and to assess their quality and suitability as an indicator of environmental pollution through direct comparison with Polish honey samples. This comparative study not only serves as an environmental monitoring tool but also aims to evaluate the potential of Algerian honey to meet European safety standards for international trade.

## 2. Results

**Figure 1:**
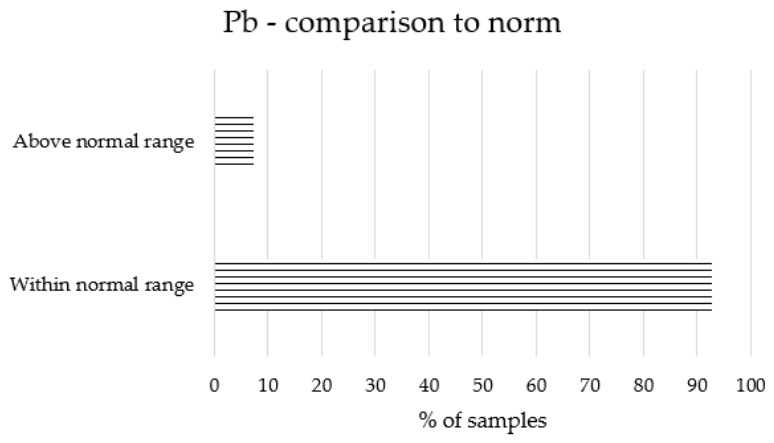
Comparison of Pb concentrations in honey with international regulatory standards.

The obtained results regarding the content of Cd, Pb, Hg and As in bee honeys are presented in Table 1 and Figure 1.

### 2.1. Comparison of As, Cd, Hg and Pb Content in Bee Honey

Honeys from Algeria exhibited a significantly higher median As concentration than those from Poland (17.615 vs. 1.570 µg/kg, *p* < 0.001). Conversely, a significantly higher median Pb content was observed in honeys from Poland (60.811 vs. 20.371 µg/kg, *p* < 0.001) (Table 1).

The observed standard deviations reflect the inherent natural heterogeneity of honey and the non-uniform distribution of trace elements associated with microscopic particles (such as pollen or dust), a phenomenon frequently reported in apicultural research [25,26]. Regarding the content of the analysed elements in individual honey varieties, no significant differences were found.

### 2.2. Comparison to Standards

The levels of toxic elements were compared with international safety standards to evaluate the quality and compliance of the honey samples. In accordance with Regulation (EU) 2023/915 [27], the maximum permissible level (MPL) for Pb in honey is fixed at 100 µg/kg. As illustrated in Figure 1, exceedances of this regulatory limit were recorded in three honey samples, all of which originated from Poland.

With regard to Cd, As, and Hg, specific maximum permissible levels for honey are not currently established within the existing European Union legislation. Consequently, the safety of these elements was evaluated by comparing the analytical results with the toxicological reference values—specifically the Tolerable Weekly Intake (TWI)—used to calculate the Estimated Weekly Intake (EWI), as detailed in the assessment of consumption safety (Section 2.3).

### 2.3. Assessment of Consumption Safety

Toxic elements such as As, Cd, Hg, and Pb are recognised for their documented toxicity and their propensity to accumulate in the human body. Therefore, in addition to comparing the analytical results with regulatory standards, it is crucial to calculate indicators to assess long-term exposure.

To evaluate consumer exposure to toxic elements through honey consumption, the estimated daily intake (EDI) and estimated weekly intake (EWI) indices were calculated. These quantify the amount of a given element consumed through the diet, determined based on the consumer’s body weight, the elemental concentration in the product, and the declared intake level. The EDI and EWI provide a starting point for further health risk assessment, as they allow for the comparison of actual exposure with reference values established by food safety bodies.

The mean EDI and EWI for As were found to be higher for honeys from Poland (0.016 ± 0.060 mg/day and 0.112 ± 0.419 mg/week, respectively). Similarly, higher values were observed for Polish honeys regarding Cd (0.037 ± 0.081 mg/day and 0.256 ± 0.566 mg/week) and Pb (0.245 ± 0.368 mg/day and 1.7171 ± 2.574 mg/week). In contrast, values for Hg were comparable for honeys from both countries (0.004 ± 0.003 mg/day and 0.030 ± 0.019 mg/week for Poland vs. 0.004 ± 0.003 mg/day and 0.031 ± 0.022 mg/week) (Table 2). Although these indicators are considered classic assessment parameters, they remain widely used in the literature. Additional indicators, which are gaining increasing importance in current toxicological research, were also calculated.

The assessment of the health safety of honey consumption from Algeria and Poland incorporated exposure indicators related to reference values established for toxic elements—the percentage of tolerable weekly intake (%PTWI) and the percentage of the reference dose determined based on dose–response modelling (%BMDL). The %PTWI reflects the share of the estimated intake of a given element from honey consumption relative to the tolerable weekly intake—defined as the amount that can be consumed throughout life without significant health risk.

Low %PTWI, %PTMI and %BMDL values indicate a small contribution of honey consumption to the total exposure to the toxic element. Values close to or exceeding 100% may indicate a significant contribution of the tested product to toxicological exposure and a potential health risk. Exposure indices remained notably low, with all values falling below 1%. Specifically, honey samples from Poland exhibited slightly higher Cd levels (%PTMI: 0.0006 ± 0.0014), whereas higher Hg values were associated with honeys from Algeria (%PTWI: 0.0276 ± 0.0200). In the case of %BMDL calculated for both As and Pb, honeys from Poland exhibited similar values (0.0028 ± 0.0104 from Poland vs. 0.0023 ± 0.039 from Algeria) or higher levels (0.0070 ± 0.0105 from Poland vs. 0.0024 ± 0.0017 from Algeria) (Table 2).

To assess the potential health risk associated with chronic exposure to toxic elements, the Target Hazard Quotient (THQ) was calculated. This dimensionless indicator is used in non-carcinogenic risk assessments and describes the relationship between the estimated level of exposure to a given element and the reference dose considered safe for the human body (RfD). The value of this indicator allows for the assessment of whether long-term consumption of a product poses a risk to the consumer. It is assumed that a THQ < 1 indicates no significant health risk, while a THQ ≥ 1 may suggest the possibility of adverse health effects resulting from long-term exposure. In the present study, the calculated indices were significantly below 1: 7.52 × 10^−7^ ± 2.29 × 10^−6^ for As, 4.83 × 10^−7^ ± 9.42 × 10^−7^ for Cd, 2.12 × 10^−7^ ± 1.46 × 10^−7^ for Hg, and 2.59 × 10^−6^ ± 4.23 × 10^−6^ for Pb (Table 3).

The Hazard Index (HI) is calculated as the sum of the THQ indices for all analysed toxic elements. An HI below 1 indicates a negligible total (cumulative) non-carcinogenic risk, while an HI of 1 or above indicates a possible non-carcinogenic risk under long-term exposure. The total mean HI value obtained was 4.00 × 10^−6^ ± 4.89 × 10^−6^ (Table 3).

### 2.4. Chemometric Analysis

Cluster Analysis (CA) was performed as part of the chemometric evaluation to categorise the samples based on the similarity of their toxic element profiles. This method groups objects into clusters whereby objects within the same group exhibit greater similarity to each other than to objects from different groups. The single-link agglomeration method was utilised, with Euclidean distance as the dissimilarity measure.

**Figure 2:**
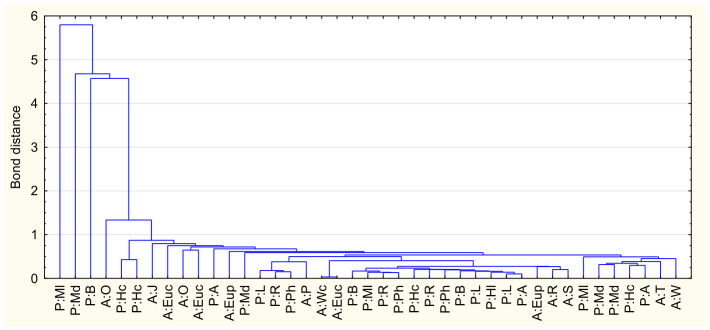
Dendrogram for bee honeys, based on the content of toxic elements (P:MI—Poland: multifloral light, P:Md—Poland: multifloral dark, P:B—Poland: buckwheat, A:O—Algeria: orange, P:Hc—Poland: honeydew coniferous, A:J—Algeria: jujube, A:Euc—Algeria: eucalyptus, P:A—Poland: acacia, A:Eup—Algeria: euphorbia, P:L—Poland: lime, P:R—Poland: rape, P:Ph—Poland: phacelia, A:R—Algeria: rosemary, A:S—Algeria: Spanish thistle, A:T—Algeria: thyme, A:W—Algeria: watercress, A:Wc—Algeria: wild carrot).

The cluster analysis revealed the presence of several distinct clusters reflecting the varying levels of contamination. Most honey samples formed compact groups that coalesced at low linkage distances, indicating similar and relatively low levels of the elements tested. A few samples integrated into the dendrogram structure only at higher distances, suggesting a different contamination profile and elevated levels of selected toxic elements. This grouping confirms the diversity in the safety profiles of the tested samples in terms of toxic element content (Figure 2).

**Figure 3:**
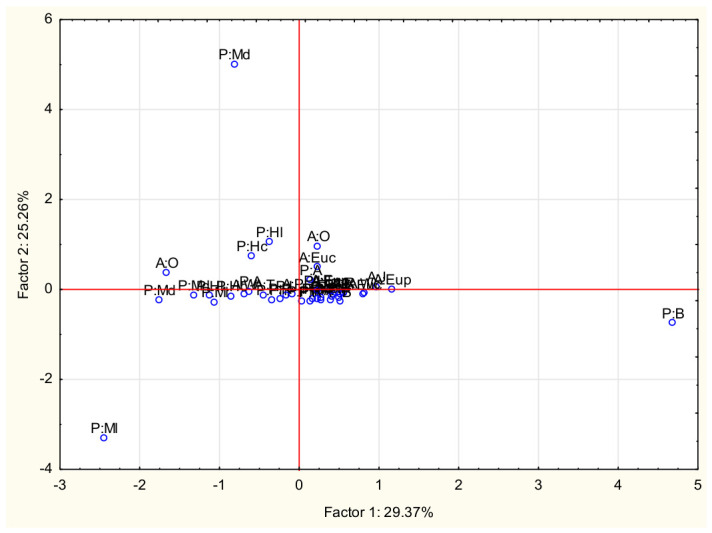
2D graph of factorial coordinates for cases (P:MI—Poland: multifloral light, P:Md—Poland: multifloral dark, P:B—Poland: buckwheat, A:O—Algeria: orange, P:Hc—Poland: honeydew coniferous, A:J—Algeria: jujube, A:Euc—Algeria: eucalyptus, P:A—Poland: acacia, A:Eup—Algeria: euphorbia, P:L—Poland: lime, P:R—Poland: rape, P:Ph—Poland: phacelia, A:R—Algeria: rosemary, A:S—Algeria: Spanish thistle, A:T—Algeria: thyme, A:W—Algeria: watercress, A:Wc—Algeria: wild carrot).

The second analysis performed was Principal Component Analysis (PCA). The first principal component (Factor 1) explained approximately 29.37% of the total variance, the second (Factor 2) accounted for 25.26%, and the third represented 24.1%. The eigenvectors indicated that the variable As exhibited a high coefficient value for the first component (0.66), while Cd showed a strong correlation with the second (0.85). Interestingly, Hg was primarily associated with the fourth component (0.69), suggesting that its distribution pattern in the studied honeys differs from that of the other toxic elements, likely reflecting a distinct source of environmental input or a specific bioaccumulation mechanism (Figure 3).

## 3. Discussion

Honey, as a natural food product with documented biological properties, should be characterised not only by high nutritional value but also by rigorous health safety. This study assessed the safety of consuming honeys from Poland and Algeria in terms of their As, Cd, Pb, and Hg content.

Natural honeys from Algeria and Poland are characterised by low levels of toxic element contamination. Exceedances of maximum residue limits (MRLs) were recorded for Pb content in only 7% of samples—all of which were from Poland. The maximum allowable content, in accordance with EU regulations, is 0.10 mg/kg for Pb, while for the remaining elements, maximum allowable levels in this type of sample are not specified. The lack of precise standards, however, does not mean that this product should not be subject to inspection and safety assessment. Honey is frequently treated as a bioindicator of environmental contamination [23]. In this context, the inclusion of samples from Poland and Algeria, representing distinct Central European and North African environmental backgrounds, allows for a comprehensive assessment. This geographical contrast is essential to validate the consistency of the calculated risk indices (EDI, EWI, PTWI, PTMI, and BMDL) across different pedoclimatic conditions and industrial histories.

Exceedances of the maximum limits established for Pb in these samples may be related to environmental mechanisms. For example, Pb strongly binds to airborne particulate matter and can settle on plant parts before entering the hive. Kędzierska-Matysek et al. [28] assessed, among other things, the content of four toxic elements in honeys (*n* = 30) from southeastern Poland. The authors noted the lowest content in honeydew honeys (0.044 mg/kg), and the highest in linden honeys (0.080 mg/kg) and rapeseed honeys (0.081 mg/kg). Among the samples tested, exceedances were noted in one rapeseed honey sample (0.107 mg/kg), two linden honey samples (0.114 and 0.158 mg/kg), and one multifloral honey sample (0.191 mg/kg). In our research, the highest value (0.756 mg/kg) was recorded for light multifloral honey.

Our research indicates that no exceedances of Cd content were observed in honeys from either geographical region. Cd content can be high in regions characterised by well-developed industry, intensive agriculture, and soils with specific geochemistry; consequently, Cd is often used for environmental profiling. Mititelu et al. observed lower values when examining honeys from southeastern Poland (*n* = 30). All samples were below the LOQ, with only one sample having a level of 0.0215 mg/kg. In our study, the highest value recorded was 0.155 mg/kg [23].

Hg is typically present in honey at trace levels. Safety assessment analyses do not indicate this product as being a major source of exposure. Significant exposure generally occurs from marine products such as fish and algae. Levels in products depend primarily on background atmospheric conditions and local emissions, rather than on a single technological factor. In a study published by Fischer et al. [29], the Hg content ranged from 0.01 to 1.71 µg/kg. The highest value was recorded in multifloral honeys (mean: 0.54 + 0.53, median: 0.33 µg/kg). The highest value recorded in our study was 4.636 µg/kg. The highest median value was found in light multifloral honeys in our study (3.468 µg/kg, mean: 2.982 ± 1.396), but no statistically significant differences were noted between varieties. Although certain identified botanical sources, such as *Fagopyrum esculentum* or *Sinapis alba*, are recognised for their metal accumulation potential, the evidence suggests that regional environmental factors and atmospheric deposition exert a more dominant influence on the final mineral profile than the botanical origin itself.

While total As content was determined, recent reports indicate that identifying chemical forms possesses higher diagnostic value, as speciation determines toxicity. For example, Jakkielska et al. [30] assessed the content of As(III), As(V), DMA, MMA, and AsB using HPLC-ICP-MS. Arsenic concentrations in honey samples ranged from 0.12 to 13 μg/kg. Interestingly, inorganic forms of As, which are more toxic, dominated in honey samples from Poland. Our study showed that in 10 honey samples from Algeria, the As content was below the detection limit. However, the median As content was significantly higher in honeys from Algeria compared to honeys from Poland (17.615 vs. 1.570 μg/kg).

The significantly higher As concentrations observed in Algerian samples (*p* < 0.001) potentially correlate with specific regional geological substrata or the localised historical use of arsenical pesticides in North African agrosystems [27], contrasting with the European context where such substances are strictly regulated [31]. Conversely, the elevated Pb levels in Polish honey samples likely reflect the persistence of legacy pollutants in soil profiles, a consequence of historical heavy industrial emissions and prolonged exposure to leaded petrol exhaust in Central Europe [28,32]. Higher As levels in Algeria are likely lithogenic (natural/soil-based) rather than anthropogenic, which highlights the natural character of the Algerian terroir. These findings underscore the necessity of site-specific risk assessments that account for regional environmental heterogeneity and soil-to-plant transfer dynamics [33,34].

Overall, the divergence between the two regions reflects a contrast between natural geochemical signatures in Algeria and the persistent industrial legacy in Poland. The presence of toxic elements in honey can be explained by various factors. For example, it depends on the location of apiaries; those situated near cities, high-traffic roads, and industrial zones will be exposed to pollen deposition from aerosols.

The dietary exposure assessment considered the EDI and EWI indices, as well as risk derivatives such as %PTWI, %BMDL, THQ, and HI. The obtained values indicate that the consumption of the tested honeys, both from Poland and Algeria, is safe and does not pose any carcinogenic or non-carcinogenic risk. Similar observations have also been reported in other publications. For example, Jakkielska et al. [30], who calculated exposure indicator values, also demonstrated that they were at levels that do not raise health concerns.

It should be emphasised that even when maximum permissible levels are exceeded in individual samples, exposure indicator values often do not indicate a health risk for the population. Health risks typically only occur for individual consumers, especially with long-term exposure cumulative with exposure from other sources. Therefore, a varied diet, based on high-quality products from reputable sources, is crucial.

The use of chemometric methods allows for a more in-depth interpretation of the obtained analytical results and the identification of relationships between the tested samples. In particular, cluster analysis facilitates the grouping of honey samples with similar toxic element profiles, aiding in the identification of those characterised by relatively higher concentrations of the analysed toxic elements. This allows for the identification of outlier samples and can serve as an additional tool supporting the assessment of the quality and safety of bee products. At the same time, it should be emphasised that the interpretation of chemometric analysis results largely depends on the number and diversity of samples. Increasing the number of analysed honey samples from different geographical regions of Poland and Algeria would enhance data variability. In the present analysis, the outlier variable, forming a separate cluster, was light multifloral honey.

Principal component analysis enables data dimensionality reduction and the identification of key factors responsible for the variability of the studied parameters. This method allows for the simultaneous analysis of multiple variables and then the representation of their relationships in the form of several principal components, which explain the largest portion of the total data variance. This allows for a visual representation of similarities and differences between the honey samples tested and the identification of which elements most significantly influence the variability. This analysis can also indicate potential relationships between samples and the levels of individual elements, enabling the identification of samples with similar chemical profiles. In the present work, the elements that explained the variability were shown to be As, Cd and Hg. The use of chemometric methods, such as cluster analysis and principal component analysis, is widely used in studies of mineral composition. The literature emphasises that these methods can be useful both in assessing the quality of honeys and in studies of their geographic and botanical origins [35].

The study also has its limitations. First and foremost, the number of honey samples analysed, particularly from Algeria, was relatively limited, which may impact the range of observed data variability and the ability to fully interpret the statistical analysis results. Increasing the number of samples, encompassing honeys of different botanical and geographical origins, would allow for greater data diversity.

## 4. Materials and Methods

### 4.1. Materials

The study included honey samples from two countries: Poland and Algeria. The honeys were obtained from retail outlets in different regions. To reduce the risk of secondary contamination, the samples were stored in the original manufacturer’s packaging. Until analysis, the samples were stored at approximately 18 °C, in a shaded area, away from direct sunlight. Prior to analysis, the samples were homogenised by thorough mixing.

### 4.2. Microwave Mineralisation in a Closed System

All honey samples were weighed to approximately 0.2–0.3 g (with an accuracy of 0.001 g) and placed in polytetrafluoroethylene (PTFE) vessels for digestion. Four mL of spectrally pure, concentrated (69%) HNO_3_ (Tracepur, Merck, Darmstadt, Germany) were then added. Microwave digestion was performed in a closed system (Berghof, Speedwave, Eningen, Germany). The process comprised four steps:

Phase 1: 170 °C, pressure: 20 atm, 10 min, 90% power;

Phase 2: 190 °C, pressure: 30 atm, 10 min, 90% power;

Phase 3: 210 °C, pressure: 40 atm, 10 min, 90% power;

Phase 4: 50 °C, pressure: 40 atm, 18 min, 0% power.

After the digestion process, the samples were quantitatively transferred to polypropylene vessels and then diluted 10-fold.

### 4.3. Determination of Cadmium, Lead, and Arsenic Content

The determination of Cd and Pb content was performed using inductively coupled plasma mass spectrometry (ICP-MS, NexION 300D, PerkinElmer, Waltham, MA, USA) in standard mode, while As was analysed using a kinetic energy discrimination (KED) cell. The results were obtained in counts per second (cps) and subsequently converted to concentrations based on calibration curves. To determine the limit of detection (LOD), 10 independent blank tests were performed; the LOD was defined as three times the standard deviation (SD) of the mean blank value. The LOD values were 0.019 μg/kg for As, 0.017 μg/kg for Cd and 0.16 μg/kg for Pb.

### 4.4. Determination of Mercury Content

Hg content was determined by atomic absorption spectrometry (AAS) using the amalgamation technique on an atomic absorption spectrometer (AMA-254, Leco Corp, Altec Ltd., Prague, Czech Republic). This mercury analyser was preferred over ICP-MS to eliminate potential memory effects and to prevent analyte loss due to the high volatility of mercury, thereby ensuring superior analytical precision. Approximately 0.02 g of honey was weighed into a nickel cuvette, and the exact mass was recorded.

The Hg content measurement comprised three stages.

Stage I: Sample combustion at 600 °C in oxygen for 60 s.

Stage II: Hg vapour was passed through a catalytic column into the amalgamator; time: 150 s.

Stage III: After release from the amalgamator, Hg content was measured at a wavelength of 254 nm for 45 s.

The detection limit was 0.003 ng/sample. Each determination was performed in triplicate.

### 4.5. Comparison with Standards

The obtained results were compared with established regulatory standards. For Pb, the maximum allowable level was defined as 100 µg/kg [27].

### 4.6. Assessment of the Safety of Honey Consumption

The PTWI value for Hg was set at 4 µg/kg BW/week, in accordance with the JECFA guidelines. Additionally, the PTMI for Cd was 25 µg/kg BW/month.

The safety of consuming natural honey was evaluated based on the following:

EDI—estimated daily intake:

EDI = C × Cons, where C is the concentration in the sample and Cons is the average level of consumption of honey in the population.

CR—cancer risk:

CR = (Fr × D × EDI × Sf)/T × 10^−3^, where Fris the frequency of exposure (365 days/year), D is the duration of exposure (70 years), EDI is the estimated daily intake, Sf is the slope factor (1.5 mg/kg/day for As, 6.3 mg/kg/day for Cd, and 0.0085 mg/kg/day for Pb), and T is the average time of exposure.

THQ—target hazard quotient:

THQ = (Fr × D × Cons × C)/(RfD × BW × T) × 10^−3^, where Fr is the frequency of exposure (365 days/year), Dis the duration of exposure (70 years), Cons is the average level of consumption, C is the concentration in the sample, RfD is the oral reference dose (0.3 µg/kg body weight/day for As, 1 µg/kg body weight/day for Cd, 1 µg/kg body weight/day for Pb, and 0.3 µg/kg body weight/day for Hg), BW is the body weight, and Tis the average time of exposure.

HI—hazard index [35,36]:

HI = ∑ (THQ_As_ + THQ_Cd_ + THQ_Hg_ + THQ_Pb_).

The calculations assumed honey consumption of 1 kg per year (because average consumption in Poland is estimated at 1 kg, and data on consumption in Algeria are currently unavailable). Consumption in some European countries is reported to be 50–70% higher.

### 4.7. Quality Control

Quality control was performed prior to the analyses and every 10 samples. A certified reference material (a mixture of Polish herbs (INCT-MPH-2)) was utilised (Institute of Nuclear Chemistry and Technology, Warsaw, Poland). The obtained results were compared with the certified values provided by the manufacturer [37]. The recoveries for As, Cd, Pb and Hg were 104%, 104%, 97.5% and 103.5%, respectively, with precision values of 2.3%, 2.5%, 3.2%, and 2.8%.

### 4.8. Statistical Analysis

The results regarding the content of toxic elements in honey samples from the two countries were analysed using Statistica 13 software (TIBCO Software Inc., Palo Alto, CA, USA). The normality of the data distribution was assessed using the Shapiro–Wilk test. Since the data followed a non-normal distribution, the Mann–Whitney U test was employed to demonstrate differences in toxic element content between honeys from the two countries, while the Kruskal–Wallis analysis of variance (ANOVA) was utilised to compare honey varieties. Statistically significant differences were categorised into three levels: *p* < 0.05, *p* < 0.01, and *p* < 0.001. Results are presented as the median and the interquartile range (Q1–Q3). To facilitate comparisons with previous studies, the mean and minimum-maximum range are also provided. Chemometric analyses were also performed, including cluster analysis, principal component analysis (PCA), and correspondence analysis.

## 5. Conclusions

The analyses conducted allow us to conclude that the content of toxic elements in honeys from Poland and Algeria, in most cases, does not exceed the applicable permissible limits specified in legal regulations. The results indicate that these honeys can be considered safe from a consumer perspective. However, it should be emphasised that population exposure is cumulative; toxic elements can enter the body through various products and various routes of exposure. Therefore, even low concentrations present in a single product, during long-term consumption, can contribute to negative health consequences. Consequently, further monitoring of toxic element content in foods of natural origin is essential in the context of long-term consumer exposure. In conclusion, this transcontinental study demonstrates that while the current toxicological burden remains within safety thresholds for the majority of samples, the observed inter-regional variability necessitates continuous monitoring. The findings highlight the global relevance of this comparative framework in harmonising food safety standards. Future research should integrate multi-isotopic fingerprinting to definitively source-track these contaminants, ensuring both consumer protection and the integrity of honey as a sensitive sentinel of environmental health. Such advanced analytical approaches will be pivotal in differentiating between natural lithogenic backgrounds and anthropogenic inputs in future apicultural studies.

## Figures and Tables

**Table 1:** Concentrations of As, Cd, Hg and Pb in bee honey samples from Algeria and Poland (µg/kg).

Criterion (Sign)	*n*	As (µg/kg)	Cd (µg/kg)	Hg (µg/kg)	Pb (µg/kg)
	Av ± SD (Min.–Max.) Med. (Q1–Q3)				
Algeria—total (A)	14	16.926 ± 4.472	9.552 ± 11.884	1.610 ± 1.170	30.154 ± 21.306
		(10.984–21.487)	(1.119–39.521)	(0.234–4.636)	(11.515–77.216)
		17.615 ^A/P^ ***	4.147	1.189	20.371 ^A/P^ ***
		(13.832–20.019)	(1.890–10.733)	(0.989–2.063)	(16.016–32.749)
Poland—total (P)	27	5.824 ± 21.852	13.324 ± 29.522	1.584 ± 0.997	89.503 ± 134.197
		(0.019–115.00)	(2.624–154.562)	(0.427–4.070)	(38.422–755.853)
		1.570 ^A/P^ ***	5.570	1.195	60.811 ^A/P^ ***
		(0.661–2.808)	(3.034–8.622)	(0.744–2.155)	(55.453–69.097)
Watercress (1)	1	<dL	1.119	3.066	11.515
Thyme (2)	1	<dL	2.115	2.616	20.248
Spanish Thistle (3)	1	<dL	1.694	1.247	16.648
Eucalyptus (4)	3	18.551	11.335 ± 12.030	1.239 ± 0.620	33.667 ± 20.933
			(1.7567–24.837)	(0.649–1.886)	(18.280–57.505)
			7.409	1.182	25.217
			(1.757–24.837)	(0.649–1.886)	(18.280–57.505)
Orange tree (5)	2	16.680	32.617 ± 9.764	2.813 ± 2.579	72.064 ± 7.286
			(25.712–39.521)	(0.989–4.6360)	(66.912–77.216)
			32.617	2.813	72.064
			(25.712–39.521)	(0.989–4.636)	(66.912–77.216)
Spurge (6)	2	10.985	6.240 ± 0.547	0.715 ± 0.680	26.621 ± 8.666
			(5.853–6.627)	(0.234–1.196)	(20.494–32.749)
			6.240	0.715	26.622
			(5.853–6.627)	(0.234–1.196)	(20.494–32.749)
Jujube (7)	1	21.487	10.733	1.081	30.451
Parsley (8)	1	<dL	1.890	2.063	16.016
Wild carrot (9)	1	<dL	2.021	0.643	15.102
Rosemary (10)	1	<dL	2.441	1.048	13.802
Acacia (11)	3	1.354 ± 1.737	10.448 ± 9.947	1.352 ± 1.004	72.194 ± 32.225
		0.019–3.318	(2.958–21.734)	(0.719–2.510)	(44.789–107.695)
		0.724	6.651	0.827	64.099
		(0.019–3.318)	(2.958–21.734)	(0.719–2.510)	(44.789–107.695)
Phacelia (12)	3	0.245 ± 0.391	3.718 ± 1.612	1.202 ± 0.643	69.507 ± 11.995
		(0.019–0.696)	(2.624–5.570)	(0.566–1.851)	(57.722–81.701)
		0.019	2.962	1.190	69.097
		(0.019–0.696)	(2.624–5.570)	(0.566–1.851)	(57.722–81.702)
Buckwheat (13)	3	40.305 ± 64.695	5.803 ± 2.794	0.709 ± 0.345	72.748 ± 3.789
		(2.877–115.009)	(3.034–8.622)	(0.427–1.094)	(68.690–76.193)
		3.030	5.752	0.607	73.362
		(2.877–115.009)	(3.034–8.622)	(0.427–1.094)	(68.690–76.193)
Lime (14)	3	1.705 ± 1.021	4.486 ± 1.484	1.078 ± 0.593	51.648 ± 14.401
		(0.661–2.701)	(2.773–5.401)	(0.640–1.753)	(38.422–66.991)
		1.753	5.284	0.841	49.531
		(0.661–2.702)	(2.773–5.401)	(0.640–1.753)	(38.422–66.991)
Rape (15)	3	0.918 ± 1.557	3.037 ± 0.523	1.126 ± 0.606	60.274 ± 5.787
		(0.019–2.716)	(2.714–3.640)	(0.488–1.695)	(55.145–66.548)
		0.019	2.757	1.195	59.128
		(0.019–2.716)	(2.714–3.640)	(0.488–1.695)	(55.145–66.548)
Honeydew conifer (16)	3	2.756 ± 0.093	14.228 ± 14.917	1.895 ± 1.046	60.617 ± 2.772
		(2.650–2.824)	(5.586–31.452)	(0.744–2.786)	(58.742–63.800)
		2.794	5.645	2.155	59.308
		(2.650–2.824)	(5.586–31.452)	(0.744–2.786)	(58.7412–63.800)
Honeydew broadleaf (17)	3	1.921 ± 0.774	17.635 ± 17.694	1.978 ± 1.100	53.566 ± 12.171
		(1.384–2.808)	(5.538–37.942)	(0.950–3.138)	(39.775–62.803)
		1.570	9.424	1.847	58.120
		(1.384–2.808)	(5.538–37.942)	(0.950–3.138)	(39.775–62.803)
Multifloral light (18)	3	0.902 ± 0.767	54.457 ± 86.702	2.982 ± 1.396	57.137 ± 3.577
		(0.019–1.400)	(3.206–154.562)	(1.408–4.070)	(53.667–60.812)
		1.288	5.601	3.468	56.933
		(0.019–1.400)	(3.206–154.562)	(1.408–4.070)	(53.667–60.812)
Multifloral dark (19)	3	2.309 ± 0.851	6.100 ± 2.875	1.933 ± 0.878	307.831 ± 389.034
		(1.356–2.992)	(3.222–8.972)	(1.093–2.844)	(55.453–755.853)
		2.579	6.107	1.861	112.188
		(1.356–2.992)	(3.222–8.972)	(1.093–2.844)	(55.453–755.853)
Total	41	7.256 ± 20.739	12.036 ± 24.813	1.593 ± 1.044	69.237 ± 112.540
		(<dL–115.009)	(1.119–154.562)	(0.234–4.636)	(11.515–755.853)
		2.579	5.570	1.195	57.722
		(0.696–2.992)	(2.773–8.622)	(0.827–2.063)	(32.749–66.912)

dL—detection limit. ***—statistically significant differences at the level of *p* < 0.001. Note: Maximum permissible level for Pb is 100 µg/kg in accordance with Regulation (EU) 2023/915. For Cd (approx. 0.05–0.1 mg/kg in the literature), As, and Hg, safety was assessed based on the toxicological reference values (TWI/PTWI) detailed in Section 2.3.

**Table 2:** Mean values of toxicological indices (EDI, EWI, %PTWI/%PTMI, and BMDL_0.5_) for the studied honey groups.

Origin	Elements	EDI (mg/day)	EWI (mg/week)	PTWI (%PTWI) or PTMI (%PTMI) (mg/kg/BW/week)	BMDL_0.5_ (%BMDL) (mg/kg/BW/day)
Algeria	As	0.013 ± 0.023	0.093 ± 0.158	-	0.0001 ± 0.0001 (0.0023 ± 0.039)
	Cd	0.026 ± 0.033	0.183 ± 0.228	0.0112 ± 0.0140 (0.0004 ± 0.0006)	-
	Hg	0.004 ± 0.003	0.031 ± 0.022	0.0004 ± 0.0003 (0.0276 ± 0.0200)	-
	Pb	0.083 ± 0.058	0.578 ± 0.409	-	0.0012 ± 0.0008 (0.0024 ± 0.0017)
Poland	As	0.016 ± 0.060	0.112 ± 0.419	-	0.0001 ± 0.0003 (0.0028 ± 0.0104)
	Cd	0.037 ± 0.081	0.256 ± 0.566	0.0156 ± 0.0347 (0.0006 ± 0.0014)	-
	Hg	0.004 ± 0.003	0.030 ± 0.019	0.0004 ± 0.0003 (0.0271 ± 0.0171)	-
	Pb	0.245 ± 0.368	1.7171 ± 2.574	-	0.0035 ± 0.0053 (0.0070 ± 0.0105)
Total	As	0.015 ± 0.050	0.015 ± 0.350	-	0.0001 ± 0.003 (0.0026 ± 0.0087)
	Cd	0.033 ± 0.069	0.231 ± 0.476	0.0145 ± 0.0283 (0.0006 ± 0.0011)	-
	Hg	0.004 ± 0.003	0.031 ± 0.020	0.0004 ± 0.0003 (0.0273 ± 0.079)	-
	Pb	0.190 ± 0.308	1.328 ± 2.159	-	0.0027 ± 0.0044 (0.005 ± 0.0088)

EDI—Estimated Daily Intake, EWI—Estimated Weekly Intake, PTWI—Provisional Tolerable Weekly Intake, PTMI—Provisional Tolerable Monthly Intake, BMDL—Benchmark Dose Lower Confidence Limit.

**Table 3:** Target Hazard Quotients (THQ), Hazard Index (HI) and Carcinogenic Risk (CR) indices for toxic elements (As, Cd, Hg, and Pb) in honey samples.

	Country	As	Cd	Hg	Pb	Total
**THQ**	Algeria	6.31 × 10^−7^ ± 1.07 × 10^−6^	3.74 × 10^−7^ ± 4.64 × 10^−7^	2.10 × 10^−7^ ± 1.53 × 10^−7^	1.18 × 10^−6^ ± 8.34 × 10^−7^	-
		0–2.80 × 10^−6^	4.38 × 10^−8^–1.55 × 10^−6^	3.05 × 10^−8^–6.05 × 10^−7^	4.51 × 10^−7^–3.02 × 10^−6^	
	Poland	7.60 × 10^−7^ ± 2.85^−6^	5.22 × 10^−7^ ± 1.16 × 10^−6^	2.07 × 10^−7^ ± 1.30 × 10^−7^	3.50 × 10^−6^ ± 5.25 × 10^−6^	-
		2.48 × 10^−9^–1.50 × 10^−5^	1.03 × 10^−7^–6.05 × 10^−6^	5.57 × 10^−8^–5.31× 10^−7^	1.50 × 10^−6^–2.96 × 10^−5^	
	Total	7.52 × 10^−7^ ± 2.29 × 10^−6^	4.83 × 10^−7^ ± 9.42 × 10^−7^	2.12 × 10^−7^ ± 1.46 × 10^−7^	2.59 × 10^−6^ ± 4.23 × 10^−6^	-
		0–1.50 × 10^−5^	4.38 × 10^−8^–6.05 × 10^−6^	3.05 × 10^−8^–6.05 × 10^−7^	4.51 × 10^−7^–2.96 × 10^−5^	
**CR**	Algeria	1.99 × 10^−5^ ± 3.38 × 10^−5^	1.65 × 10^−4^ ± 2.05 × 10^−4^	-	7.02 × 10^−7^ ± 4.96 × 10^−7^	
		0–8.83 × 10^−5^	1.93 × 10^−5^–6.82 × 10^−4^		2.68 × 10^−7^–1.80 × 10^−6^	
	Poland	2.39 × 10^−5^ ± 8.98 × 10^−5^	2.30 × 10^−4^ ± 5.10 × 10^−4^	-	2.08 × 10^−6^ ± 3.13 × 10^−6^	
		7.81 × 10^−8^–4.73 × 10^−4^	4.53 × 10^−5^–2.67 × 10^−3^		8.95 × 10^−7^–1.76 × 10^−5^	
	Total	2.37 × 10^−5^ ± 7.22 × 10^−5^	2.13 × 10^−4^ ± 4.16 × 10^−4^	-	1.54 × 10^−6^ ± 2.52 × 10^−6^	
		0–4.73 × 10^−4^	1.93 × 10^−5^–2.67 × 10^−3^		2.68 × 10^−7^–1.76 × 10^−5^	
**HI**	Algeria	-	-	-	-	2.40 × 10^−6^ ± 1.91 × 10^−6^
						7.54 × 10^−7^–6.81 × 10^−6^
	Poland	-	-	-	-	4.99 × 10^−6^ ± 5.94 × 10^−6^
						2.07 × 10^−6^–3.02 × 10^−5^
	Total	-	-	-	-	4.00 × 10^−6^ ± 4.89 × 10^−6^
						7.54 × 10^−7^–3.02× 10^−5^

CR—Carcinogenic Risk, HI—Hazard Index, THQ—Total Hazard Quotient.

## Data Availability

The original contributions presented in the study are included in the article, further inquiries can be directed to the corresponding author.

## Abbreviations

The following abbreviations are used in this manuscript:

AAS	Atomic Absorption Spectroscopy
As	Arsenic
Cd	Cadmium
Pb	Lead
Hg	Mercury
ICP-MS	Inductively Coupled Plasma—Mass Spectrometry
